# Potential Role of the Resident Mesenchymal Stem-Like Cells in Renal Fibrogenesis after Ureteral Obstruction

**DOI:** 10.1155/2019/2543171

**Published:** 2019-03-05

**Authors:** Yong-Hua Peng, Jie Xiao, Chen Yan, Lan Luo, Tao-Sheng Li

**Affiliations:** ^1^Department of Nephrology, The First Affiliated Hospital of Guangzhou Medical University, Guangzhou 510120, China; ^2^Department of Stem Cell Biology, Atomic Bomb Disease Institute, Nagasaki University, 1-12-4 Sakamoto, Nagasaki 852-8523, Japan

## Abstract

The mechanisms of renal fibrogenesis after ureteral obstruction remain unclear. We tried to primarily expand mesenchymal stem cells from renal tissues and then investigated their role in fibrogenesis after ureteral obstruction. Unilateral ureteral obstruction was induced by ligating the left ureteral duct of adult C57BL/6 mice. We collected the kidneys for experiments at 2, 7, and 14 days after operation. Histological analysis showed obviously fibrotic changes in the left kidney at 7 days and further increased at 14 days after ureteral obstruction. To expand mesenchymal stem cells, we minced the renal tissues into small explants (about 1 mm^3^) and cultured onto 10 cm dishes. Interestingly, the outgrowth of cells was observed significantly earlier from the explants of the obstructed left kidney than that of the unobstructed right kidney. These expanded cells showed the potency of adipogenic, osteogenic, and chondrogenic differentiations and positively expressed with CD44 and partly expressed with CD90, CD105, and CD106, but negatively expressed with CD34, CD45, and FSP1, suggesting the phenotype of mesenchymal stem-like cells (MSLCs). The mouse fibrosis RT^2^ profiler PCR array showed that many genes were changed over 2-fold in the MSLCs expanded from both kidneys at 2, 7, and 14 days after operation. Interestingly, profibrotic genes were prevalently enhanced in the left kidney with ureteral obstruction. Histological analysis also showed obviously infiltration of inflammatory cells in the left kidney at 14 days after operation. Our data indicate the potential role of resident MSLCs in renal fibrogenesis after ureteral obstruction, but further experiments are required to understand the relevant mechanisms.

## 1. Introduction

Fibrosis, a common histological feature for chronic kidney diseases, can be induced by various pathological conditions, such as mechanical obstruction, toxins, infections, and autoimmune diseases [1]. As the deposition of pathological matrix in the interstitial space and within the walls of glomerular capillaries is known to accelerate kidney injury, it is critical to prevent and ameliorate the pathological fibrogenesis for kidney disease treatment.

Actually, fibrogenesis is generally recognized as a common procedure of repair/regeneration of tissues/organs in response to various injuries. Fibroblasts are well known to synthesize stress fiber and to deposit extracellular matrix [2]. Myofibroblasts and leukocytes have also been demonstrated to involve in the process of fibrogenesis [3]. Otherwise, pericytes/perivascular cells of kidney peritubular capillaries have recently been confirmed as matrix-forming cells [1]. However, the cellular and molecular mechanisms of renal fibrogenesis have not yet been completely understood.

In past decades, renal progenitor cells [4] and mesenchymal stem cells [5] have been identified in the adult mammalian kidney. These resident stem/progenitor cells are known to play critical roles in maintaining the homeostasis of the kidneys. Stem/progenitor cells of renal origin have also showed the ability to engraft into the damaged kidneys [6], mitigate functional loss [7], and generate nephrons *de novo* [8], suggesting the potential applications for curing renal diseases. However, few studies have investigated the role of resident stem/progenitor cells on the process of renal fibrogenesis. Because the repair/regeneration of the injured organs is always accompanied with fibrogenesis, it is quite possible that the resident stem/progenitor cells, especially the mesenchymal stem cells, play potential role in renal fibrogenesis under pathological conditions.

Experimental model of unilateral ureteral obstruction (UUO) has provided extensive information on renal fibrogenesis [9]. Past studies have documented solid evidence of fibrotic lesions in the kidneys at 7 days after ureteral obstruction [9]. By using the well-established UUO model in healthy mice, we tried to expand mesenchymal stem cells from renal tissues at different times after operation. We observed dynamic changes on the number and biological characterizations of mesenchymal stem-like cells (MSLCs) after mechanical obstruction, including the enhanced expression of profibrotic genes, which indirectly suggested the likely role of resident MSLCs on renal fibrogenesis.

## 2. Materials and Methods

### 2.1. Experimental Animals

Adult (9-14 weeks old) male C57BL/6 mice (CLEA Japan Inc.) were used for the experiments. This study was approved by the Institutional Animal Care and Use Committee of Nagasaki University (no. 1108120943-8). All animal procedures were performed in accordance with the institutional and national criteria and recommendations.

### 2.2. Unilateral Ureteral Obstruction (UUO) Model

The UUO model was established by an identical surgical procedure as previously described [10]. Briefly, general anesthesia was induced to the mice by intraperitoneal injection with pentobarbital sodium (60 mg/kg). A flank incision was used to expose the left kidney and ureter. The left ureter was ligated with 5-0 silk suture. At 2, 7, and 14 days after operation, the animals were sacrificed and the left kidneys were extracted for following experiments (the obstructed group, *n* = 18). The right unobstructed kidneys were also extracted to serve as biologic controls (the unobstructed group, *n* = 18). The kidneys from sham-operated animals were used as negative controls (the sham group, *n* = 6).

### 2.3. Masson's Trichrome Staining

To detect the fibrotic changes, 6 *μ*m thick cryosections were made from the frozen renal tissues embedded in Tissue-Tek optimal cutting temperature compound (Sakura, Tokyo, Japan) as previously described [11]. Masson's trichrome staining was performed according to the manufacturer's protocol (Sigma-Aldrich, St. Louis, MO, USA). The stained sections were mounted and then imaged with a microscope (Biorevo BZ-9000; Keyence Japan, Osaka, Japan). Fibrotic area was quantified by calculating the positive-stained area using the Image-Pro Plus software (version 5.1.2, Media Cybernetics Inc., Carlsbad, CA, USA), and the mean fibrotic area of 9 randomly selected images from three separated slides was used for statistical analysis.

### 2.4. Ex Vivo Expansion of MSLCs from Renal Tissues

The *ex vivo* expansion of MSLCs from renal tissues was done as previously described [12]. In brief, freshly extracted renal tissues were minced into approximately 1 mm^3^ fragments and cultured as “explants” on 10 cm dishes (30 fragments/dish) coated with 15 *μ*g/ml fibronectin (Corning) solution. Stromal-like flat cells grew out from the explants at almost 3-5 days and became confluent at approximately 16 days. We harvested the outgrowth cells using 0.25% trypsin (Gibco/Life Technologies, Grand Island, NY, USA) at 14 days after the initiation of culture. The collected cells were counted using a NucleoCounter cell-counting device (ChemoMetec A/S, Denmark). Cells were further expanded by cell passage, and twice-passaged cells were used for further analyses. All cells were maintained in IMDM medium (Gibco) supplemented with 10% fetal bovine serum (HyClone) and 1% penicillin/streptomycin (Gibco) and cultured at 37°C in a humidified atmosphere of 5% CO_2_ and 95% air.

### 2.5. Assessment of Cell Proliferation

Cell proliferation was evaluated by the MTT [3-(4,5-dimethylthiazol-2-yl)-2,5-diphenyltetrazolium bromide] assay as previously described [13]. Briefly, twice-passaged MSLCs were seeded in 96-well culture plates at a density of 1 × 10^4^ cells/well. After 48 hours culture, MTT was added to the medium. The formation of formazan from MTT in viable cells was stopped by adding lysis buffer 24 h after the addition of MTT. The absorbance of formazan was measured at 570 nm using a microplate reader (Thermo Scientific, Multiskan FC, USA).

### 2.6. Flow Cytometry and Immunocytochemistry

The characterization of *ex vivo* expanded cells from renal tissues was investigated by flow cytometry as previously described [14]. Briefly, twice-passaged cells were incubated with FITC or PE-conjugated antibodies against CD4, CD34, CD44, CD45, CD73, CD90, CD106, and MHC II (eBioscience), CD105 (Abcam), and FSP1 (Millipore), for 30 minutes. Isotype-identical antibodies served as negative control. Quantitative analysis was performed using a FACSCalibur flow cytometer with CellQuest software (Becton Dickinson). Differentiation of the twice-passaged cells into adipocytes, osteocytes, and chondrocytes was also examined by using the Mouse Mesenchymal Stem Cell Functional Identification Kit (R&D Systems, Minneapolis, USA), according to the manufacturer's instructions.

### 2.7. Mouse Fibrosis RT^2^ Profiler PCR Array

To analyze the expression of genes related to fibrosis, we purified RNA from the expanded cells by using ISOGEN II (NIPPON GENE). The concentration of RNA was determined using a NanoDrop 2000 spectrophotometer (Thermo Fisher Scientific), and 1 *μ*g of RNA was used to generate cDNA using the RT^2^ First Strand Kit (SABiosciences, a Qiagen Company). The mouse fibrosis RT^2^ Profiler PCR array was performed according to the manufacturer's instructions (#330231 PAMM-120ZA, SABiosciences). This PCR array profiles 84 key genes involved in dysregulated tissue remodeling during the repair and healing of wounds. The fold change of expression was calculated using the SABiosciences web-based data analysis program. The results represent the mean expression of three independent samples.

### 2.8. Immunofluorescence Staining

To estimate the infiltration of inflammatory cells in the renal tissues, immunostaining was conducted on the renal tissues. Briefly, frozen sections were fixed with 4% formalin (Wako) for 10 min at room temperature. After blocking, tissue sections were incubated with rat anti-mouse CD86 antibody (1 : 200 dilution, eBioscience), rat anti-mouse F4/80 antibody (1 : 200 dilution, eBioscience), and goat anti-mouse CD11c antibody (1 : 200 dilution, Abcam) overnight at 4°C. After washing with PBS, tissue sections were incubated with Alexa Fluorescent 488-conjugated anti-goat Ig (1 : 200 dilution) and Alexa Fluorescent 594 anti-rat Ig (1 : 200 dilution) secondary antibodies at room temperature for 1 hour in the dark. The immunofluorescence was examined under microscope (Olympus).

### 2.9. Statistical Analysis

All data were presented as the mean ± SD. Statistical analysis was performed by one-way ANOVA and followed by Tukey's multiple comparison test (Dr. SPSS II, Chicago, IL). A *p* value less than 0.05 was accepted as significant.

## 3. Results

### 3.1. Renal Fibrosis Was Clearly Induced by Ureteral Obstruction

**Figure 1:**
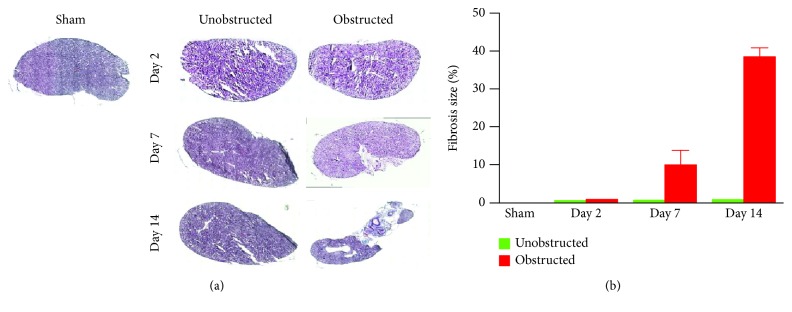
Masson's staining for renal fibrosis. (a) Representative images of renal tissues. (b) Quantitative data on the fibrotic area in each group.

As shown in the representative images of Masson's trichrome staining (Figure 1(a)), fibrotic changes were extensively observed in the left kidney at 7 and 14 days after ureteral obstruction. In contrast, fibrotic tissues were rarely observed in the right kidney without ureteral obstruction. Quantitative data showed that the fibrotic area was significantly higher in the obstructed group than the unobstructed group at 7 days (10.08 ± 2.21% vs. 0.78 ± 0.04%, *p* = 0.013; Figure 1(b)) and 14 days (38.54 ± 1.35% vs. 0.88 ± 0.02%, *p* < 0.001; Figure 1(b)) after operation.

### 3.2. Ureteral Obstruction Accelerated the Outgrowth of Mesenchymal Stem-Like Cells (MSLCs) from Renal Tissues

**Figure 2:**
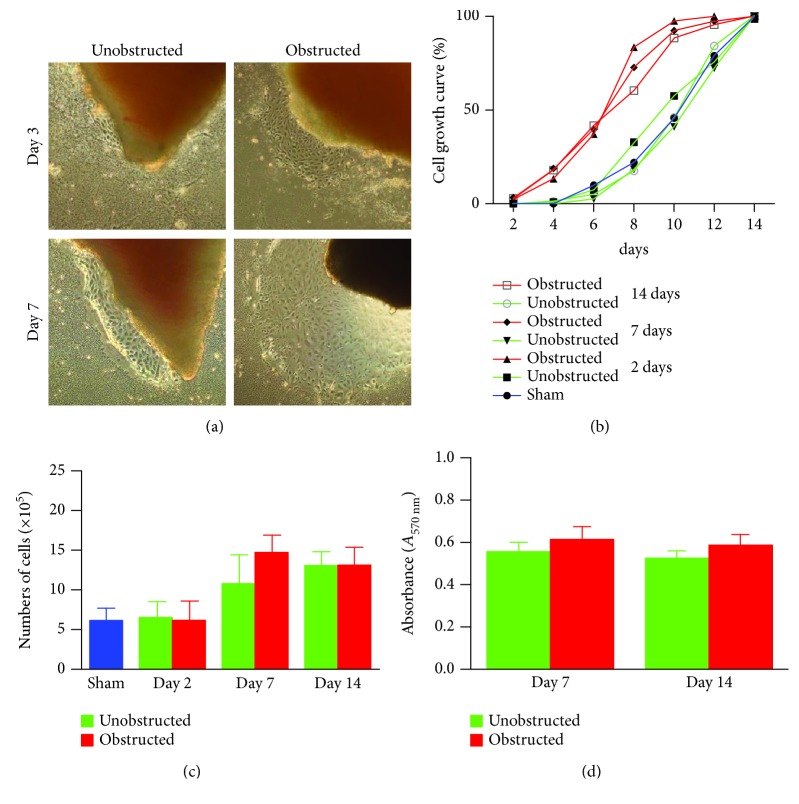
Dynamics of cell outgrowth from renal tissues by culturing as “explants.” (a) Representative images show the outgrowth of cells from the “explants” of renal tissues at 3 and 7 days after planting on the dish. (b) The curve lines represent the percentages of “explants” with cell growth at different times (days) after the initiation of culture in each group. (c) The numbers of outgrowth cells harvested at 14 days after the initiation of culture. (d) MTT assay on the proliferation of twice-passaged cells.

Although the outgrowth of cells from explants was mostly observed at 3-5 days after culture (Figure 2(a)), some explants from the obstructed left kidneys could clearly see the outgrowth of cells even at 1 day after the initiation of culture. By plotting the observed time of cell outgrowth from all explants, we could find that the cell outgrowth was significantly earlier in the obstructed group than the unobstructed or sham groups (*p* < 0.01, Figure 2(b)). We harvested and counted the outgrowth cells at 14 days after culture. Compared with the healthy kidney in the sham group, the number of outgrowth cells in either of the obstructed group or the unobstructed group was significantly higher at 7 and 14 days (*p* < 0.01, Figure 2(c)), but not at 2 days after operation (Figure 2(c)). The total number of outgrowth cells was not significantly different between the obstructed group and the unobstructed group, although the time of cell outgrowth from explants was observed earlier in the obstructed group. Otherwise, MTT assay also showed that the proliferation of twice-passaged MSLCs was not significantly different between the obstructed group and the unobstructed group (Figure 2(d), *p* = 0.099).

### 3.3. Characterizations of Ex Vivo Expanded MSLCs from the Kidneys

**Figure 3:**
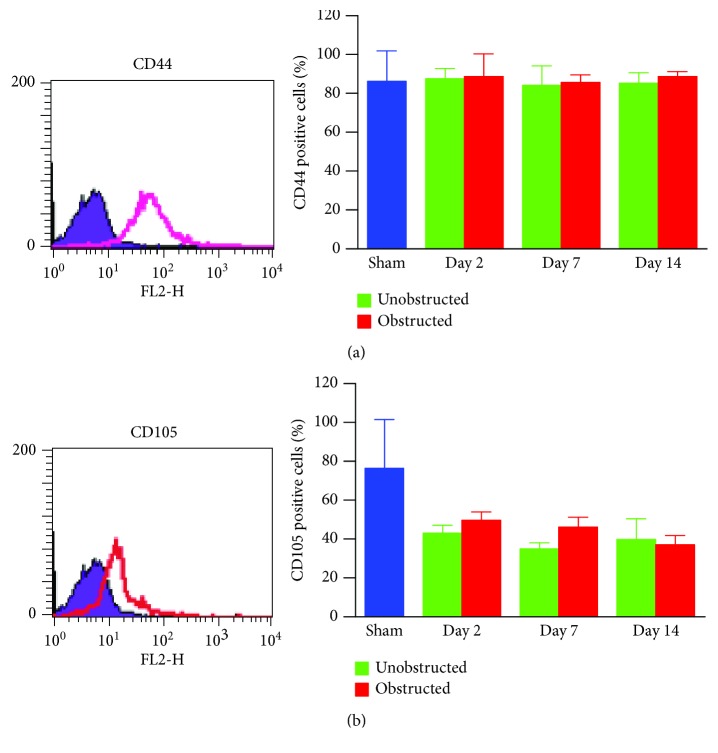
Expression of CD44 and CD105 in the ex vivo expanded cells from renal tissues. Twice-passaged cells are used for measuring the expression of CD44 (a) and CD105 (b) by flow cytometry. The representative histograms (left) and quantitative data (right) are shown.

**Figure 4:**
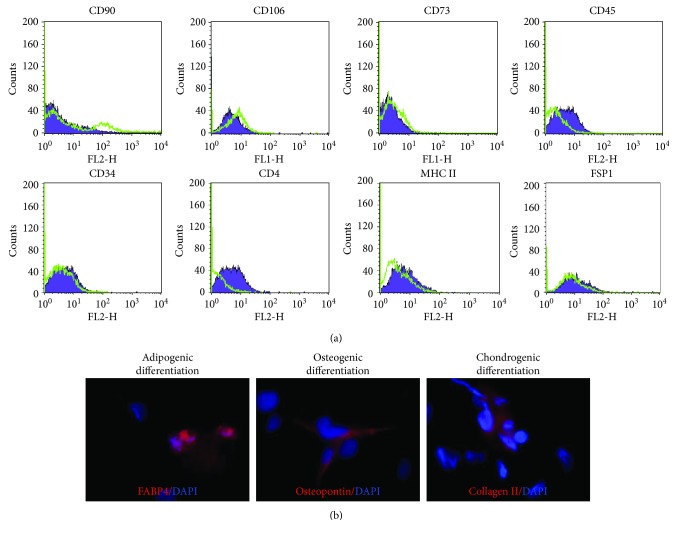
Phenotype characterization of *ex vivo* expanded cells. (a) Representative histograms of flow cytometry analysis show the expressions of CD90, CD106, CD73, CD45, CD34, CD4, MHC II, and FSP1 in the twice-passaged cells expanded from renal tissues at 7 days after ureteral obstruction. (b) Representative images of immunofluorescent staining on the adipogenic (FABP4-positive, red), ostogenenic (osteopontin-positive, red), and chondrogenic (collagen II-positive, red) differentiations from the twice-passaged cells. Nuclei are counterstained with 4′,6-diamidino-2-phenylindole (DAPI, blue).

These *ex vivo* expanded MSLCs from renal tissues of all groups were positively expressed with CD44 (>90%) (Figure 3(a)), partly expressed with CD105 (35-50%) (Figure 3(b)), CD90 (6-43%), CD106 (3-10%), and CD73 (2-8%), but negatively expressed with CD34, CD45, CD4, MHC II, and FSP1 (Figure 4(a)). *In vitro* assay confirmed the potency of adipogenic, osteogenic, and chondrogenic differentiations from the twice-passaged cells (Figure 4(b)), suggesting the phenotype of mesenchymal stem-like cells.

### 3.4. Ureteral Obstruction Prevalently Enhanced the Expression of Profibrotic Factors in MSLCs

**Figure 5:**
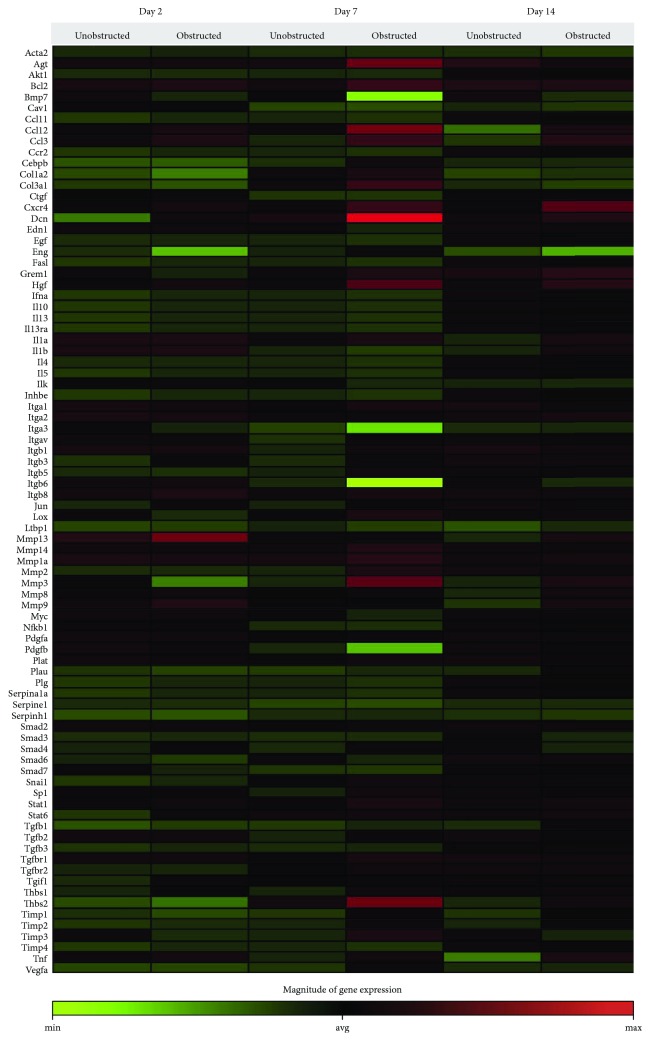
Overall changes on the expression of genes related with fibrosis in the *ex vivo* expanded cells from renal tissues. The heat map shows the overall changes of expression of genes including in the mouse fibrosis RT^2^ profiler PCR array. Twice-passaged cells are used for analysis, and the expression levels are comparing to the sham group.

**Figure 6:**
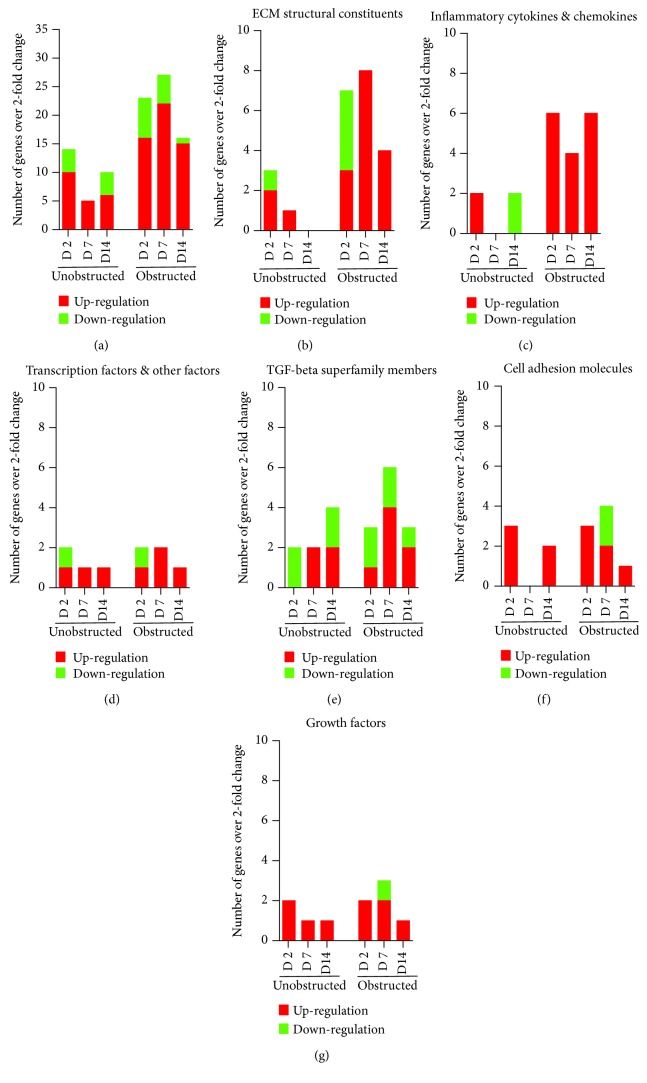
The number and pattern of fibrosis genes that up- and downregulated over 2-fold in the *ex vivo* expanded cells from renal tissues. (a) Total numbers of genes that up- and downregulated over 2-fold in the twice-passaged MSLCs of the unobstructed and obstructed groups when comparing to the sham group. According to the biological functions, these genes with over 2-fold change are also categorized into ECM structural constituents and remodeling enzymes (b), inflammatory cytokines and chemokines (c), transcription factors and other factors (d), TGF-beta superfamily members (e), cell adhesion molecules (f), and growth factors (g).

Data from the mouse fibrosis RT^2^ profiler PCR array showed an extensive change on the expression of genes related to fibrogenesis in these MSLCs expanded from either the obstructed left kidney or the unobstructed right kidney at 2, 7, and 14 days after surgery, as compared with the healthy kidney from mice received sham operation (Figure 5). However, more genes were up- or downregulated over 2-fold at 2, 7, and 14 days in the obstructed group than those in the unobstructed group, and the largest difference between groups was observed at 7 days after operation (Figure 6(a)). We further categorized these genes according to their biological functions. Interestingly, these genes that functionally related to the ECM structural constituents (Figure 6(b)), inflammatory cytokines and chemokines (Figure 6(c)), and TGF-beta superfamily members (Figure 6(e)) were voluminously changed in these MSLCs expanded from the obstructed left kidney at 2, 7, and 14 days after operation.

**Figure 7:**
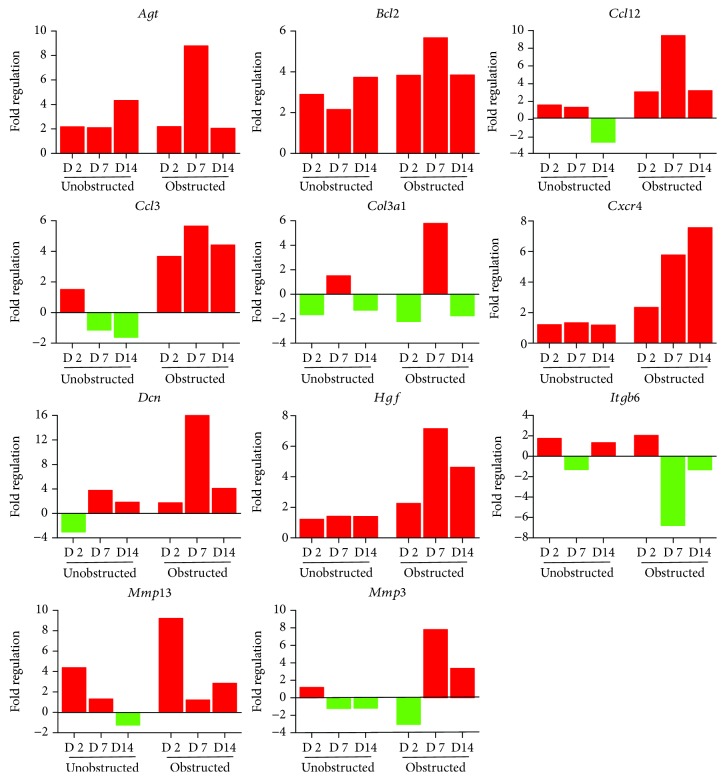
The fold changes of expression of representative genes. Data shows the representative genes that up- and downregulated over 5 folds in the renal tissues at any time point after unilateral ureteral obstruction.

By listing these genes with over 5-fold change at any time point after operation, we noticed that the majority of these genes encoding with profibrotic factors were predominantly changed in these MSLCs expanded from the obstructed left kidney (Figure 7). Otherwise, many of these genes, such as *Agt*, *Ccl12*, *Col3a1*, *Dcn*, *Itgb6*, and *Thbs2*, were temporarily changed in these MSLCs expanded from the left kidney at only 7 days after ureteral obstruction, but the changes of *Ccl3* and *Cxcr4* were constantly observed during the 14 days of follow-up (Figure 7). The predominantly change on the expression of profibrotic factors suggested a probable role of MSLCs in renal fibrogenesis in response to mechanical obstruction.

### 3.5. The Infiltration of Inflammatory Cells in Renal Tissues

**Figure 8:**
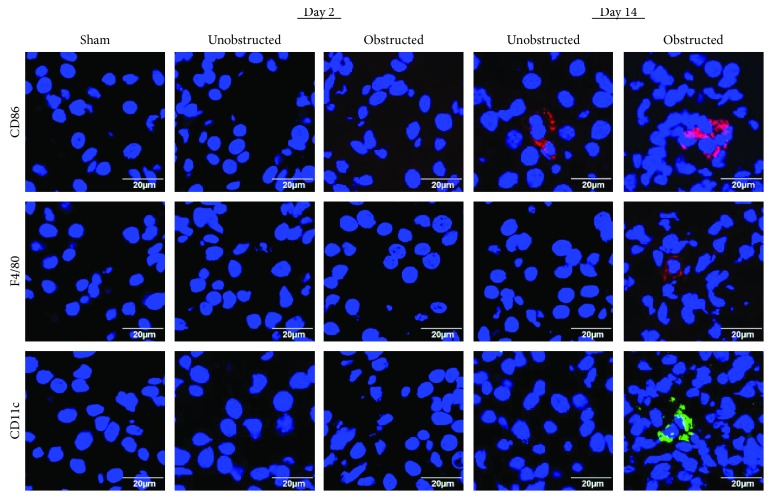
Infiltration of inflammatory cells in renal tissues. Representative images of immunofluorescence staining with CD86, F4/80, and CD11c show the infiltration of inflammatory cells in renal tissues. Nuclei are counterstained with 4′,6-diamidino-2-phenylindole (DAPI, blue).

The infiltration of inflammatory cells in renal tissues was detected by immunofluorescence staining (Figure 8). Representative images showed that the expressions of CD11c and F4/80 were highlighted in the left kidney with ureteral obstruction at 14 days after operation, but the expression of CD86 was observed in both the obstructed left kidney and the unobstructed right kidney. The expression of Ly6G was detected negatively in renal tissues from all groups (data not shown).

## 4. Discussion

Experimental and clinical studies have demonstrated that mesenchymal stem cells of different origins accelerate the repair/regeneration of damaged tissues/organs with a reduced scar formation [5, 15]. However, mesenchymal stem cells express abundantly with both of prefibrotic and antifibrotic genes [16], suggesting the dual faces of mesenchymal stem cells in fibrosis. Using the UUO model to induce renal fibrosis, this study was purposed to investigate the potential role of resident mesenchymal stem cells in fibrogenic process under pathological condition.

Several methods, such as microdissection or enzymatic digestion, have been used to isolate the renal stem/progenitor cells from the normal kidneys [17, 18]. In 2006, Plotkin and Goligorsky have successfully isolated a clone of 4E cells from the adult murine kidneys [17]. These 4E cells express positively with CD44, CD73, and Sca-1 and show the characteristics similar to mesenchymal renal embryonic cells with multiple differentiation potency of mesodermal lineages, including fibroblasts and pericytes, which are known to involve in fibrogenic process. Many previous studies have also confirmed CD44 as a marker for mesenchymal stem cells of renal origin [18, 19]. However, there has few of study on the purification/expansion of stem cells from damaged kidney tissues [20].

Agreed well with past studies [16, 17], we confirmed that ureteral obstruction induced extensively renal fibrosis within 1 week in adult healthy mice. Two methods are popularly used to achieve primary mesenchymal stem cells from various solid tissues/organs. One is directly culturing the tissues on dishes to grow mesenchymal stem-like cells as described in this study and another is to enzymatically digest the tissues into single-cell suspension and then purify mesenchymal stem cells for expansion. However, it is still the absence of consensus on the cell surface marker(s) for purifying mesenchymal stem cells. As it is difficult to get enough renal tissues from patients and then digest into single-cell suspension for cell sorting, we selected to expand MSLCs by culturing renal tissues. By culturing the renal tissues as small “explants” on dishes, we could easily expand enough number of cells from renal tissues of mice received either sham operation or unilateral ureteral obstruction surgery. These expanded cells from renal tissues positively expressed CD44, a popular marker for mesenchymal stem cells of renal origin [18, 19], and also fairly expressed with another mesenchymal stem cell marker of CD105. As CD90 and CD73 are only the golden markers for identifying mesenchymal stem cells from human rather than from mouse [21], it was not surprised about the poorly/negatively expression of CD90 and CD73 in these expanded cells from renal tissues of mouse. However, these twice-passaged cells from renal tissues showed the potency of multipotent differentiations and negative expressed with fibroblast specific marker of FSP1, which suggests a phenotype of mesenchymal stem-like cells. Interestingly, compared with the kidney from sham-operated mice, unilateral ureteral obstruction induced a dynamic change on the number and biological characterization of resident MSLCs primarily expanded from both kidneys.

It keeps unclear how unilateral ureteral obstruction changes the number and biological characterization of MSLCs even in the contralateral kidney without ureteral obstruction. The outgrowth of cells was observed earlier from explants of the obstructed kidney than that of the unobstructed kidney, although the proliferative activity was not significantly different between groups. This might be related with the different levels of cytokines/chemokines between groups. Previous studies have demonstrated that UUO causes a sustained glomerular ischemia by activating the renin angiotensin system [22], and renal ischemia is known to stimulate the proliferation of stem cells [23]. Ureteral obstruction may also increase the expression of TGF-*β* to induce the epithelial mesenchymal transition of tubular epithelial cells [24]. Otherwise, in response to injury, interstitial cells are required to support the regeneration of the kidneys by assuming an immature phenotype [25]. Therefore, it was not surprising about the dynamic changes of resident MSLCs in both kidneys after unilateral ureteral obstruction.

Many genes related to fibrogenesis were up- and downregulated over 2-fold in these MSLCs ex vivo expanded from both kidneys after unilateral ureteral obstruction. The changed expression of some genes in the MSLCs from the unobstructed right kidney might be due to a pathophysiological response to various stresses, such as surgical injury and the change of blood flow in the kidneys. Although some genes, such as *Ccl3* and *Cxcr4*, changed persistently, the changes of many genes were temporarily observed after unilateral ureteral obstruction. We noticed that the expressions of profibrotic factors, including the ECM structural constituents, inflammatory cytokines and chemokines, and TGF-beta superfamily members, were prevalently changed in these MSLCs *ex vivo* expanded from the left kidney with ureteral obstruction. The ECM structural constituents and TGF-beta superfamily members are well known as direct players/regulators of fibrogenesis. The enhanced expression of inflammatory cytokines and chemokines, such as *Ccl3* and *Cxcr4*, can recruit inflammatory cells to participate the fibrogenic process. Actually, many F4/80- and CD11c-positive cells were observed in the left kidney with ureteral obstruction. *In vitro* experiments, such as the cocultures of MSLCs with macrophages and lymphocytes [26], may help to understand whether the resident MSLCs activate inflammatory cells to induce the renal fibrosis after ureteral obstruction [21]. Although further studies are asked to demonstrate the casually relationship between the changes of biological characterizations of resident MSLCs and the renal fibrosis, it is quite possible that mechanical obstruction activates the resident MSLCs to induce profibrotic effect in the left kidney.

This study has several limitations. First, these ex vivo expanded MSLCs were a heterogeneous population and showed different patterns on the expression of cell surface markers as compared to the mesenchymal stem cells from bone marrow and adipose tissue. Although the expression of CD44 is quite similar among groups, we did not try to purify the CD44-positive cells for comparison about their gene expression profiling. Second, we did not provide direct evidence on the correlation between the gene expression changes in MSLCs and the renal fibrosis after ureteral obstruction. Third, it is well known about the similarity on biological characteristics between mesenchymal stem cells and fibroblasts. Although the negative expression of FSP1 in the *ex vivo* expanded MSLCs suggested a different phenotype with fibroblasts, it will be better to isolate fibroblasts from the same renal tissues for comparison in the study. Otherwise, it is highly required to confirm the up- and down-regulated genes at a protein level and further identify the key factors of regulating the renal fibrosis after ureteral obstruction.

## 5. Conclusions

We successfully expanded MSLCs from the kidneys using a simple method of tissue explant culture and found that unilateral ureteral obstruction induced dynamic changes on the number and biological characterizations of resident MSLCs in both kidneys. The enhanced expression of profibrotic genes in these MSLCs from the obstructed left kidney indirectly suggests the potential role of resident MSLCs in renal fibrosis after ureteral obstruction. The regulation of resident MSLCs may provide novel approach for the prevention and treatment of renal fibrosis.

## Data Availability

The data used to support the findings of this study are available from the corresponding author upon request.
